# The Role of Diffusion-Weighted Magnetic Resonance Imaging in the Differentiation of Head and Neck Masses

**DOI:** 10.3390/jcm7060130

**Published:** 2018-05-29

**Authors:** Lutfi Kanmaz, Erdal Karavas

**Affiliations:** 1 Department of Otorhinolaryngology—Head and Neck Surgery, Pazarcık State Hospital, Kahramanmaraş 46700, Turkey; lutfikanmaz@gmail.com; 2 Department of Radiology, Faculty of Medicine, Erzincan University, Erzincan 24100, Turkey

**Keywords:** neck mass, diffusion-weighted MRI, apparent diffusion coefficient

## Abstract

The purpose of this study was to evaluate the value of diffusion-weighted MRI (DW-MRI) in differentiating benign and malignant head and neck masses by comparing their apparent diffusion coefficient (ADC) values. The study included 32 patients with a neck mass >1 cm in diameter who were examined with echo planar DW-MRI. Two different diffusion gradients (b values of b = 0 and b = 1000 s/mm^2^) were applied. DWI and ADC maps of 32 neck masses in 32 patients were obtained. Mean ADC values of benign and malignant neck lesions were measured and compared statistically. A total of 15 (46.9%) malignant masses and 17 (53.1%) benign masses were determined. Of all the neck masses, the ADC value of cystic masses was the highest and that of lymphomas was the lowest. The mean ADC values of benign and malignant neck masses were 1.57 × 10^−3^ mm^2^/s and 0.90 × 10^−3^ mm^2^/s, respectively. The difference between mean ADC values of benign and malignant neck masses was significant (*p* < 0.01). Diffusion-weighted MRI with ADC measurements can be useful in the differential diagnosis of neck masses.

## 1. Introduction

Quick and accurate diagnosis directly affects treatment success for patients with a neck mass, which is a common finding in ENT clinics. Inadequate or late diagnosis of a malignant mass increases the morbidity and mortality of a disease.

The rapid development of diagnostic imaging technology has provided clinical practice with new facilities for the evaluation of neck masses. These new methods are gaining importance with the advantageous factors of cost and ease of use. At present, ultrasonography (USG) and/or computed tomography (CT) are used as conventional methods for the evaluation of neck lesions.

If necessary, magnetic resonance imaging (MRI) is used for the characterization of neck masses. MRI evaluates the morphology, signal intensity and enhancement pattern of lesions. However, none of these methods can accurately differentiate benign from malignant lesions. This has led to the necessity of researching new diagnostic methods. Diffusion-weighted magnetic resonance imaging (DW-MRI) is a non-contrast enhanced technique that can be obtained during a single breath-hold. In the literature, DW-MRI was first used in the early diagnosis of stroke in neuroradiology [1,2]. In the early period, the use of this technique was limited in the central nervous system due to its sensitivity to cardiac, respiratory, and peristaltic movements. However, following improvements in the echo planar imaging technique as a fast MRI sequence, it became possible to successfully apply diffusion-weighted echo planar MRI even in other areas with high-susceptibility artifacts. DW-MRI was first applied to head and neck lesions in 2001 and promising results have been achieved [3]. Subsequent studies showed that DW-MRI appeared to be helpful in differentiating epidermoid carcinoma and malignant lymphoma, staging neck nodal disease, and distinguishing radiotherapy-induced tissue changes from persistent or recurrent cancer. In these studies, apparent diffusion coefficient (ADC) values of tissues and lesions are calculated using diffusion-weighted images and different values in the differential diagnosis. Moreover, with the use of this imaging technique, the creation of an ADC map is an excellent method for differentiation between the viable and necrotic parts of head and neck tumors. Thus, the ADC map can be used to select the best biopsy site and to detect tumor viability in the post-treatment follow-up of patients after radiation therapy. The technique may also be useful in characterizing thyroid nodules and salivary gland neoplasms.

The purpose of this study was to evaluate the value of DW-MRI in differentiating benign and malignant head and neck masses by comparing their ADC values.

## 2. Materials and Methods

This prospective study was performed on 43 consecutive patients who underwent MRI for a diagnosis of head and neck lesions in our center. All patients were examined with contrast-enhanced MRI and DW-MRI. The study was conducted in the Department of Otorhinolaryngology, Bakırköy Dr. Sadi Konuk Training and Research Hospital, in the period of June 2009 to June 2010.

Institutional Ethics Committee approval was obtained for the study.

### 2.1. Subjects

A total of 11 patients were excluded from the study; four patients with neck masses <1 cm in the greatest minimal transverse diameter, two who had undergone biopsy, three due to distortion artifacts, and two with a final diagnosis of neck metastasis of a thyroid carcinoma.

Thus the final study population of 32 consecutive patients with neck masses >1 cm in diameter consisted of 12 females (37.5%) and 20 males (62.5%) with a mean age of 45.13 ± 17.08 years (range, 9–78 years). All patients were questioned in detail about age, location, and duration of the mass, associated symptoms, and then routine blood tests such as serological tests were applied. Within the head and neck examination, diagnostic pan-endoscopy of the nasal cavity, nasopharynx, oropharynx, hypopharynx, and larynx was also performed. All clinical evaluations were documented.

When patients had multiple neck masses with the same histological diagnosis, only the largest one was used for calculation of ADC values. Thus, the diffusion-weighted images and ADC maps of 32 neck masses in 32 patients were studied.

Localization of the lesions was classified according to the lymph node regions and neck facial spaces. The final diagnosis of the patients was made by histopathological examination of surgical specimens. A diagnosis of tuberculosis lymphadenitis was made by histology and culture, two undifferentiated nasopharyngeal carcinoma metastases by primary tumor biopsy and FNAB, neck metastasis in five patients with NHL diagnosed by excisional lymph node biopsy, and one adenocarcinoma metastasis by FNAB. A diagnosis of SCC metastasis was confirmed with neck dissection. Diagnosis of carotid body paraganglioma in one patient was established with MR angiography and DSA (digital subtraction angiography) before excision.

### 2.2. MR Imaging Techniques

All the MR examinations were performed with a 1.5 Tesla MR unit (Siemens Avanto, Erlangen, Germany). Routine examination consisted of T2-weighted fast spin-echo images (with a section thickness of 4 mm, an interslice gap of 1–2 mm, a field of view (FOV) of 25–30 cm and an acquisition matrix of 256 × 224) and DW-MRIs. Before DW-MRI, T2-weighted images were obtained in the axial plane. A total of 14 transverse images covering the lesions were obtained. DW-MRIs were obtained at the section level where the largest transverse section of the lesion was detected on the MRIs which were obtained before administration of contrast material. DW-MRI was obtained using multi-slice spin-echo single-shot echo planar imaging in the axial plane. For each patient, diffusion-weighted images and ADC maps were obtained by applying diffusion-sensitive gradients in three orthogonal directions (x, y, and z) and two different b-values (0 and 1000 s/mm^2^). ADC maps of the images were automatically reconstructed on the main console. Then, the region of interest (ROI) was defined by a radiologist measuring the signal intensity of the lesion. ROIs were determined on the solid appearing parts for the solid masses and on the cystic areas for the cystic lesions. The ADC values of the lesions were calculated with an ROI >1 cm^2^.

### 2.3. Statistical Analysis

Statistical analyses of the study data were performed using NCSS (Number Cruncher Statistical System) 2007 and PASS (Power Analysis and Sample Size) 2008 Statistical Software (HyLown Consulting LLC., Atlanta, GA, USA). The Student’s *t*-test was used to compare data between two groups. Results were stated as the mean and standard deviation. Qualitative data were compared using the chi-square test. The receiver operating characteristic (ROC) curve was applied to determine the cut-off point with the highest accuracy and sensitivity. The value of *p* < 0.05 was considered statistically significant at a 95% confidence level.

## 3. Results

This study was performed on 32 consecutive patients with head and neck masses who underwent echo planar DW-MRI from June 2009 to June 2010. The patients consisted of 12 females (37.5%) and 20 males (62.5%) with a mean age of 45.13 ± 17.08 years (range, 9–78 years).

Malignant masses were determined in 15 (46.9%) cases and benign masses in 17 (53.1%). The benign masses consisted of five pleomorphic adenoma originating from major salivary glands, three reactive lymphadenopathies, two branchial cleft cysts, two cervical sympathetic chain schwannomas, two Whartin’s tumors, one glomus tumor, one tbc lymphadenitis, and one thyroglossal duct cyst. The malignant masses were five Non-Hodgkin’s lymphoma, three larynx SCC met., two undifferentiated carcinoma met., two oropharynx SCC met., one GIS adeno ca met., one primary unknown carcinoma met., and one tonsil SCC met. The diagnoses of the patients are listed in Table 1.

Of the total neck masses, the ADC value of cystic masses was the highest (1.98 × 10^−3^ mm^2^/s) and that of lymphomas (0.80 × 10^−3^ mm^2^/s) was the lowest. The mean ADC values of benign and malignant neck masses were 1.57 × 10^−3^ mm^2^/s and 0.90 × 10^−3^ mm^2^/s, respectively. The difference between the mean ADC value of benign and malignant neck masses was statistically significant (*p* < 0.01). The localizations of the masses are listed in Table 1. The numbers of malignant and benign masses were 15 (46.9%) and 17 (53.1%), respectively. The mean ADC value of benign masses with high signal intensity was statistically significantly higher than that of malignant masses with low signal intensity (*p* < 0.01) (Table 2). Malignant masses were classified in two categories as malignant lymphomas (33.3%) and carcinomas (66.7%, squamous cell carcinoma or adenocarcinoma). There was no statistically significant difference between the two categories in the malignant group (*p* > 0.05) (Table 2). When an ADC value of 1.13 × 10^−3^ mm^2^/s or less was used to predict malignancy, the best results were achieved with high accuracy, with sensitivity of 93.33%, specificity of 82.35%, positive predictive value of 82.35%, and a negative predictive value of 93.33% (Table 3).

**Figure 1:**
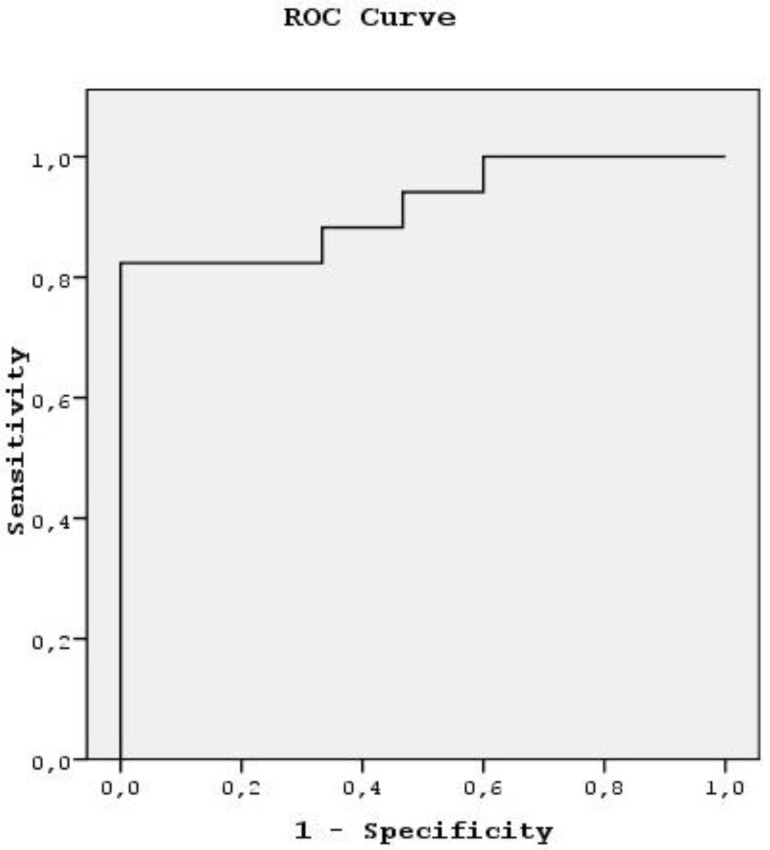
Receiver operating characteristic (ROC) curve of the ADC value.

The ROC curve was used to evaluate the diagnostic capability of the ADC value to differentiate benign from malignant masses. When 1.13 × 10^−3^ mm^2^/s was used as a threshold value in differentiating benign from malignant masses, the area under the curve was 0.918 (Table 4, Figure 1).

## 4. Discussion

Diffusion is defined as the randomized microscopic movement of water molecules and is used as a sensitive parameter for the characterization of tissue at a microscopic level. Today, in vivo measurement of diffusion is possible with DW-MRI and ADC measurements. As a result of new technological developments, MRI has become sensitive to the diffusion of water protons in biological tissues and diffusion-weighted imaging can be obtained. Intracellular and extracellular water balance is also shown in a way that is important for diagnosis and follow-up of stroke. Initially, the use of this technique was limited to brain studies because of technical problems regarding motion artifact due to cardiac, respiratory, and peristaltic movements. However, following improvements in echo planar imaging techniques, an echo planar DW-MRI can now be successfully performed even in areas with high susceptibility artifacts [4]. This technique was first used in neuroradiological imaging for diagnosis of early cerebral ischemia and has become a diagnostic tool in this area [1,5].

In 1994, Muller et al. measured the ADC of water in liver, spleen, kidney, and muscle and showed that in vivo diffusion measurements of abdominal organs obtained with MRI could prove helpful in the identification and classification of abdominal disease [6]. Subsequently, in several studies, DW-MRI has been shown to be able to be used in the differential diagnosis of lesions in the liver, kidney, and other abdominal organs with the measurement of ADCs [7,8].

In the literature, DW-MRI has also been seen to have an application area in the different regions of the head and neck. The characterization of head and neck lesions with echo planar DW-MRI by Wang et al. [3] was the first study in this area. They found that the mean ADC value of the benign lesions was statistically significantly different than that of malignant lesions. In 2003, Sumi et al. [9] studied the differential diagnosis of metastatic lymph nodes with diffusion-weighted MR imaging. Over several years, further studies have been made in this area. In another study on neck lymph nodes, ADC maps, as a new technique, have been used to determine the necrotic and non-necrotic solid areas of malignant lesions [10]. Eida et al. [11] reported that preoperative tissue characterization of salivary gland tumors could be made with ADC map construction. In another study, Abdel Razek et al. reported that the mean ADC value of malignant thyroid nodules was statistically significantly lower than that of benign ones [12]. The diffusion technique involves the diffusion motion of water protons in the tissues. According to the diffusion of tissue, the diffusion of water molecules varies in the different regions of tissue. Therefore, the diffusion coefficient of the tissue varies depending on any change in the proportion of extracellular to intracellular water molecules. Thus, diffusion-weighted MR imaging produces different contrast and ADC values according to the microstructure of the tissues [3].

**Figure 2:**
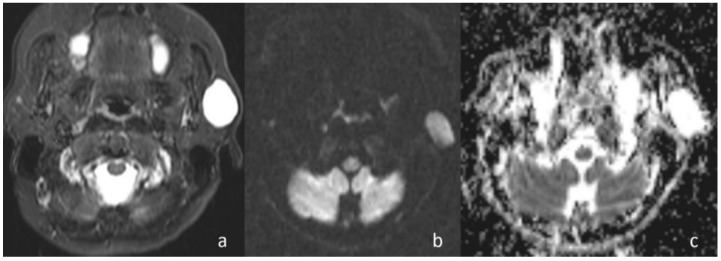
Pleomorphic adenoma of the parotid gland. (**a**) Axial T2-weighted image (T2WI) shows a mass in the left parotid gland; (**b**) the lesion shows low signal intensity on DWI; and (**c**) the mass is hyperintense on the ADC map (ADC value of 1.55 × 10^−3^ mm^2^/s).

**Figure 3:**
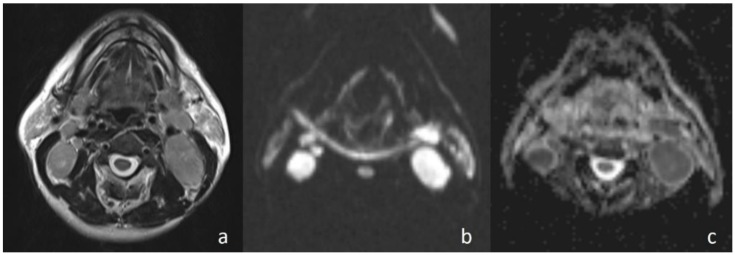
Undifferentiated nasopharyngeal carcinoma. (**a**) Axial T2WI shows bilateral metastatic cervical lymph nodes; (**b**) lymph node, on the left side of the neck, shows high signal intensity on DWI; and (**c**) the mass is hypointense on the ADC map (ADC value of 0.91 × 10^−3^ mm^2^/s).

In the current study, the mean ADC value of benign masses with high signal intensity was significantly (Figure 2) higher than that of malignant (Figure 3) masses with low signal intensity. These differences in ADC values may be explained by the differences in the histopathological characteristics of benign and malignant tumors. Generally, malignant tumors show hypercellularity and have enlarged nuclei, and hyperchromatism. These histopathological characteristics reduce the diffusion space of water protons in the extracellular and intracellular regions [13,14].

**Figure 4:**
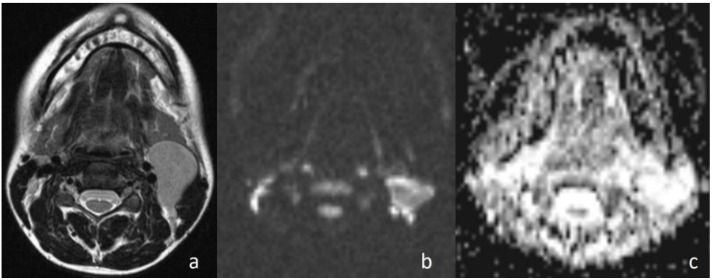
Second branchial cleft cyst. (**a**) Axial T2WI shows a unilocular cystic mass in the left carotid space; (**b**) the lesion shows low signal intensity on DWI; and (**c**) the lesion is hyperintense on the ADC map (ADC value of 2.02 × 10^−3^ mm^2^/s).

Apparently higher ADC values for benign cystic masses may be expected because of the relatively freer mobility of water protons in the fluid. In the current study, cystic masses were not grouped separately due to low numbers. However, consistent with previous studies, the mean ADC value of three cystic masses (Figure 4) (1.98 × 10^−3^ mm^2^/s) was higher than that of other benign solid masses (ADC = 1.48 × 10^−3^ mm^2^/s). In addition, the differences in ADC values among cystic masses could be explained by the different protein concentrations. A high protein level restricts the movement of water molecules by increasing the viscosity [15].

**Figure 5:**
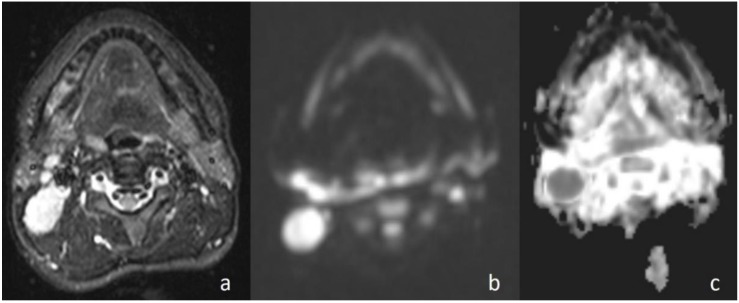
Non-Hodgkin lymphoma. (**a**) Axial T2WI shows metastatic cervical lymph node nearby SCM; (**b**) the lymph node, on the right side of the neck at level of lateral cervical region, shows high signal intensity on DWI; and (**c**) the mass is hypointense on the ADC map (ADC value of 0.62 × 10^−3^ mm^2^/s).

Sumi et al. [16] reported that the mean ADC value of lymphomas is lower than that of metastatic lymph nodes of carcinomas due to the difference in cellular density. Maeda et al. [17] also reported that carcinomas could contain small foci of necrosis on histopathological examination that was not identifiable on conventional MRI. This investigation has also been used to explain the higher ADC values of SCCs than those of lymphomas. In the current study, although there was no statistically significant difference, the mean ADC value of lymphomas (Figure 5) (0.80 × 10^−3^ mm^2^/s) was lower than that of the other malignant tumors (0.96 × 10^−3^ mm^2^/s). In the malignant group, larynx SCC metastasis with the value of 1.15 × 10^−3^ mm^2^/s showed the highest values.

One of the considerations that must be taken into account when using diffusion-weighted MRI for the differential diagnosis of masses is the recognition of the fact that some malignant masses behave like a benign tumor independently of the ‘cut-off’ ADC value obtained at the end of the study. These masses are some primary or metastatic SCC and thyroid carcinoma metastases [3]. In the current study, a case with diagnosis of larynx SCC metastasis in the ADC value of 1.20 × 10^−3^ mm^2^/s showed the feature of a benign tumor. The reason that the high ADC values are non-standardized might be the presence of small foci of micronecrosis and the hypervascular tumor portions that escalate the perfusion effect in some malignant tumors and of dense extracellular fluid in follicular components in thyroid carcinomas [3]. Considering this situation, the macroscopic solid portions were determined using MRI observations and the ADC measurements were made, and the cases with neck masses of thyroid origin were excluded from the study. Although DW-MRI is more reliable than that of other MR imaging techniques to identify micronecrosis of primary or metastatic tumors, [18,19,20] the differentiation between the viable and necrotic parts of head and neck tumors with DW-MRI by reconstructing ADC maps is possible for accurate biopsy results [21]. In addition, DW-MRI with ADC measurement may be used for differentiating residual or recurrent head and neck tumors from postoperative or postradiation changes [22].

With concern for inaccurate ADC measurements in small lesions according to the susceptibility artifacts and image distortions as a limitation of this imaging technique, neck masses <1 cm in the greatest minimal transverse diameter on MRI were excluded from the study. In a study by Chen et al., performed to compare periodically rotated overlapping parallel lines with enhanced reconstruction (PROPELLER) DW-MRI and echo planar DW-MRI techniques, it was suggested that it was possible to reduce the distortion of head and neck masses through PROPELLER diffusion-weighted MRI to a large extent [23].

One of the limitations of the current study was the area of necrosis that showed falsely higher ADC values, especially in the center of metastatic neck masses [10]. Therefore, ADC values were measured by selecting the solid portions of tumors.

**Figure 6:**
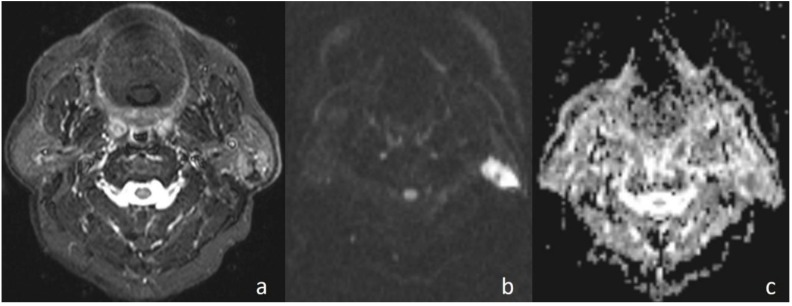
Warthin tumor. (**a**) Axial T2WI showing the mass localised in the superficial lobe and spreading into the deep lobe of the left parotid gland; (**b**) the lesion shows high signal intensity on DWI; and (**c**) the lesion is hypointense on the ADC map (ADC value of 0.88 × 10^−3^ mm^2^/s) and the tumor was falsely diagnosed as a malignant lesion.

In the study, three masses in the benign group indicated lower ADC values than the cut-off value in accordance with the malignant mass. In one of these masses, which was tuberculous lymphadenopathy (ADC = 0.92 × 10^−3^ mm^2^/s); restriction of diffusion could be explained by the presence of inflammatory cells in the pus that reduced the diffusion space of water protons [24]. In another study, this situation was explained by the thickness of the caseous material of granulomatous lesions [25]. Whartin tumor (ADC = 0.88 × 10^−3^ mm^2^/s) was the other case that was falsely diagnosed as a malignant tumor (Figure 6). Intense lymphoid accumulation in the stroma and proliferation of the epithelial component could be the reason for the limited motion of the water protons in the extracellular space [3]. Reactive lymphadenitis was the third case. A varying amount of fibrosis in the stroma of inflammatory cells could have resulted in the low ADC value by restriction of the diffusion of water molecules [3].

In the current study, in cases with malignant diseases, the mass appeared hyperintense on diffusion images (obtained at b = 1000 s/mm^2^) and with low signal intensity on ADC maps and, conversely, benign masses appeared hypointense and hyperintense, respectively. There was a statistically significant difference in ADC values between the malignant masses and benign lesions (*p* < 0.01). When an ADC value of 1.13 × 10^−3^ mm^2^/s or less was used to predict malignancy, the best results were achieved with high accuracy, with 93.33% sensitivity, 82.35% specificity, 82.35% positive predictive value, and 93.33% negative predictive value.

In this study, the patient groups were heterogeneous with different histopathological entities and there was a limited number of cases in each benign or malignant group. Further investigations of a larger series with a specific group of the same pathological diagnosis are necessary.

## 5. Conclusions

In summary, DW-MRI seems to be a promising non-invasive imaging technique for characterization of head and neck masses or for any other subjects as discussed above. It can be concluded that further studies on larger series and advances of diffusion MR techniques to improve the image quality would help DW-MRI to become a routine imaging technique.

## Figures and Tables

**Table 1:** Diagnosis and localization of 32 head and neck masses.

**Diagnosis**	***n***	**%**
Pleomorphic adenoma	5	15.6
Reactive lymphadenitis	3	9.4
Branchial cleft cyst	2	6.3
Schwannoma	2	6.3
Whartin tumor	2	6.3
Glomus tumor	1	3.1
Tbc lymphadenitis	1	3.1
Thyroglossal duct cyst	1	3.1
Non-hodgkin’s lymphoma	5	15.6
Larynx SCC metastasis	3	9.4
Undifferentiated carcinoma metastasis	2	6.3
Oropharynx SCC metastasis	2	6.3
GIS adenocarcinoma metastasis	1	3.1
Primary unknown carcinoma met.	1	3.1
Tonsil SCC metastasis	1	3.1
**Localizations**	***n***	**%**
Anterior cervical	1	3.1
Superior lateral cervical	9	28.1
Middle lateral cervical	3	9.4
Posterior cervical	1	3.1
Sup-mid lateral cervical	2	6.3
Parapharyngeal area	3	9.4
Parotid area	7	21.9
Submandibular area	5	15.6
Supraclavicular area	1	3.1

**Table 2:** Pathological diagnosis and distribution of mean ADC values in the examined groups.

Pathology	*n*	%	ADC (×10^−3^ mm^2^/s) Ave. ± SD.	+*p*
Malignant	15	46.9	0.90 ± 0.17	0.001 **
Benign	17	53.1	1.57 ± 0.42	
Lymphoma	5	33.3	0.80 ± 0.14	0.100
Carcinoma	10	66.7	0.96 ± 0.17	

+ Student *t* test; ** *p* ≤ 0.001.

**Table 3:** Calculating the threshold value.

Value	Sensitivity	Specificity	Positive Predictive Value	Negative Predictive Value	Accuracy	Relative Risk
0.95	66.67	88.24	83.33	75.00	78.13	3.33
0.98	66.67	82.35	76.92	73.68	75.00	2.92
1.05	80.00	82.35	80.00	82.35	81.25	4.53
1.12	86.67	82.35	81.25	87.50	84.38	6.50
1.13	93.33	82.35	82.35	93.33	87.50	12.35
1.20	100.00	82.35	83.33	100.00	90.63	-
1.21	100.00	76.47	78.95	100.00	87.50	-
1.34	100.00	70.59	75.00	100.00	84.38	-

**Table 4:** Area under the curve (AUC).

Area under the Curve				
Area	Std. Error (a)	*p*	95% Confidence Interval	
			Upper	Lower
0.918	0.05	0.001	0.819	1.016
